# *De novo* missense mutation in *MYT1l* leading to autosomal dominant intellectual disability 39 and autism spectrum disorder: a case report

**DOI:** 10.3389/fped.2025.1672911

**Published:** 2025-10-23

**Authors:** Xin Wang, Shuangzhu Lin, Yang Chen, Yangfan Qi, Xiaoyu Sun, Wanqi Wang, Kai Jiang

**Affiliations:** ^1^ School of Traditional Chinese Medicine, Changchun University of Chinese Medicine, Changchun, China; ^2^ Diagnosis and Treatment Center for Children, The Affiliated Hospital of Changchun University of Chinese Medicine, Changchun, China

**Keywords:** autism spectrum disorder, global developmental delay, autosomal dominant intellectual disability type 39, *MYT1l*, epilepsy

## Abstract

**Background:**

Autosomal dominant intellectual disability type 39 (MRD39; OMIM # 616521) is caused by heterozygous mutation in the *MYT1l* gene on chromosome 2p25.3. The *MYTL1* encoded protein belongs to a novel class of cystein-cystein-histidine-cystein zinc finger proteins that function in the developing mammalian central nervous system.

**Case summary:**

We report a 1-year-6-month-old girl presenting with global developmental delay (GDD) and autistic behaviors, demonstrating inability to stand independently, crawling mobility, poor response to name calling, and impaired joint attention. Initial developmental assessments yielded a Griffiths Mental Development Scale score of 57 and an ADOS-2 score of 11. Following 20 months of systematic rehabilitative training, the patient achieved independent ambulation, could follow simple commands, and produced phrases under 10 words, though suboptimal response to name calling and joint attention persisted. Re-evaluation showed a Griffiths score of 59 and an ADOS-2 score of 10. Whole-exome sequencing identified a *de novo* heterozygous missense variant in the *MYT1l* gene [c.1695G > T; p.(Arg565Ser)]. According to the American College of Medical Genetics and Genomics (ACMG) guidelines, this variant was classified as Likely Pathogenic based on criteria PM6 (*de novo* status) and PM2 (absence in population databases). Based on the concordant genotype and phenotype, the patient was diagnosed with *MYT1l*-related neurodevelopmental disorder (MRD39).

**Conclusion:**

We report a case of *MYT1l*-related disorder presenting with global developmental delay and features of autism spectrum disorder, associated with the previously documented but functionally uncharacterized c.1695G > T (p.Arg565Ser) variant. This case provides valuable clinical evidence supporting the pathogenicity of this variant and contributes to a deeper understanding of the phenotypic spectrum of *MYT1l*-related conditions.

## 1 Introduction

Autosomal dominant intellectual disability type 39 (MRD5; OMIM #616521) is caused by heterozygous mutation in the *MYT1l* gene on chromosome 2p25.3 (1). The *MYT1l* gene comprises 12 exons and encodes a protein of 1,230 amino acids (1, 2). The core clinical manifestations of MRD39 include intellectual disability, delayed language development, autism spectrum disorder, with partial patients exhibiting distinctive facial features and epileptic seizures (3–5).

In this study, we present a case of a patient diagnosed with MRD39, characterized by significant developmental delay and autistic features, yet notably lacking seizures. Whole-exome sequencing (WES) revealed a *de novo* heterozygous missense mutation in the *MYT1l* gene, which aligns with the patient's clinical phenotype. This finding contributes to the expanding knowledge of *MYT1l* variants and their associated clinical presentations.

## 2 Case presentation

### 2.1 Chief complaint

A 1-year-6-month-old girl was referred for evaluation of developmental delay observed over the preceding 8 months.

The patient presented with inability to ambulate independently since birth. This female infant was born at 37 weeks' gestation (G2P2) via spontaneous vaginal delivery, with a birth weight of 3.0 kg. There was no history of intrauterine hypoxia or neonatal resuscitation. She was formula-fed postnatally. Developmental milestones included: head control at 4 months, rolling over at 6 months, independent sitting at 11 months, stable sitting without support at 14 months, crawling at 15 months, and standing independently at 16 months. Clinical observations revealed poor response to name calling, limited eye contact, absence of stranger anxiety, and prominent repetitive head-banging behavior. Initial developmental assessments yielded a Griffiths Mental Development Scale score of 57 and an ADOS-2 score of 11.

### 2.2 Physical examination

Weight 10 kg, head circumference 46 cm. The child was alert and responsive with regular respirations. No hypopigmented patches or café-au-lait spots were observed. The anterior fontanelle was closed. Ocular motility was normal, and the pupils were isocoric with normal light reflexes. No nuchal rigidity was noted. Cardiopulmonary and abdominal examinations were unremarkable. Cranial nerve assessment showed no abnormalities. Muscle strength and tone were normal in all extremities. Knee jerks and abdominal reflexes were present. Ankle clonus was absent. Kernig's sign, Brudzinski's sign, Babinski sign, and Oppenheim sign were negative bilaterally.

### 2.3 Family history

The patient's father and mother are healthy, and the patient's 8-year-old brother is healthy.

### 2.4 Routine clinical examinations

A comprehensive evaluation, including complete blood count, urinalysis, stool analysis, assessments of liver and kidney function, cardiac enzyme levels, electrolyte balance, thyroid function, and both blood and urine genetic metabolic screenings, indicated no abnormalities. Electroencephalography (EEG) revealed no abnormalities, and brain magnetic resonance imaging (MRI) showed no structural lesions.

### 2.5 Genetic analysis

**Figure 1:**
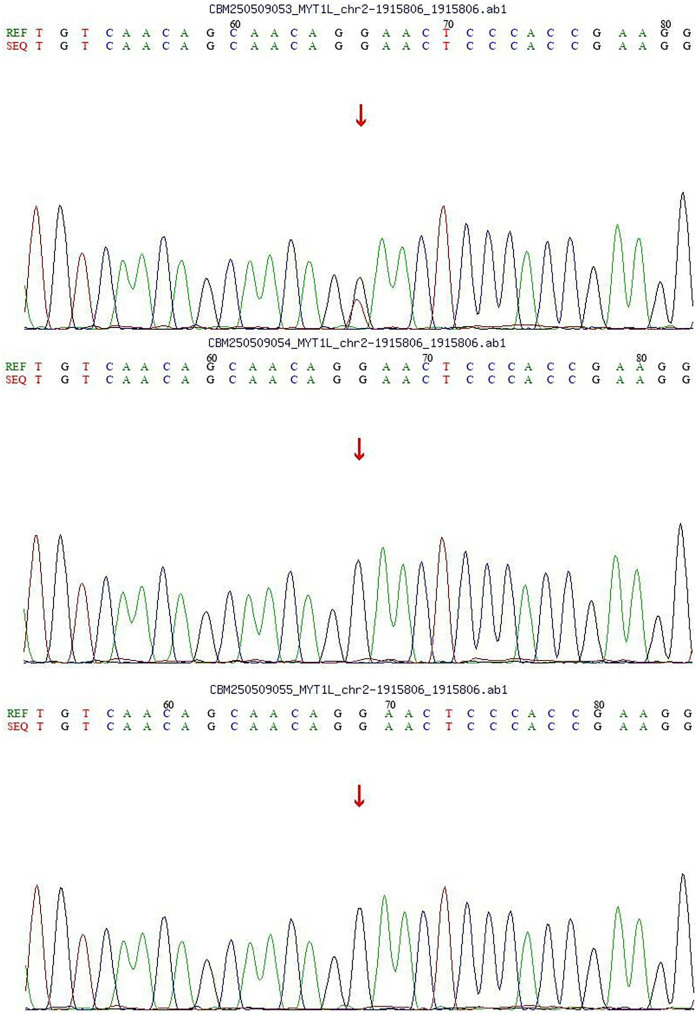
Sanger diagram.

With the consent of the parents, we performed whole exome sequencing (WES) to identify potential pathogenic variants.Following sequencing, a stepwise filtering approach was applied to prioritize candidates: common polymorphisms (allele frequency >0.1% in aggregate population databases including gnomAD, 1,000 Genomes, and ExAC) were excluded, and focus was placed on rare, protein-altering variants. Given the patient's phenotype and negative family history, we prioritized *de novo* variants. Whole-exome sequencing (WES) revealed a *de novo* heterozygous missense variant in the *MYT1l* gene [c.1695G > T; p.(Arg565Ser)]. Parental Sanger sequencing confirmed that both parents carried the wild-type allele for this variant (Figure 1). Based on the ACMG/AMP guidelines, the variant was assessed as Likely Pathogenic, supported by evidence PM6 (confirmed *de novo* status) and PM2 (absence in population databases).

### 2.6 Final diagnosis

MRD39, ASD.

### 2.7 Treatment

A comprehensive rehabilitation program spanning 20 months was implemented for the patients.

### 2.8 Outcome and follow-up

Following 20 months of systematic rehabilitation training, the patient achieved independent walking and short-distance running, and can speak short phrases of fewer than 10 words. However, persistent deficits include reduced eye contact, poor response to name calling, and stereotypical head-banging behavior. Re-evaluation showed a Griffiths score of 59 and ADOS-2 score of 10.

## 3 Discussion

The *MYT1l* gene is integral to neuronal differentiation and neural development. Studies demonstrate that *MYT1l* sustains neuronal identity by repressing the expression of genes associated with non-neuronal lineages. This repression occurs through its interaction with the Sin3b complex, which is recruited via the N-terminal domain of *MYT1l* (6). Furthermore, *MYT1l* exhibits similar genomic binding sites in both neurons and fibroblasts and is typically found in an open chromatin configuration, emphasizing its vital role in maintaining neuronal identity (6).

Beyond its function in neurons, *MYT1l* also facilitates the differentiation of oligodendrocyte precursor cells (OPCs). Evidence indicates that *MYT1l* is expressed during myelin formation and remyelination, with its expression being regulated by binding to the Olig1 promoter, thereby promoting OPC differentiation (7). These biological functions position *MYT1l* as a promising therapeutic target for demyelinating diseases.

In neurodevelopmental disorders, loss-of-function mutations in the *MYT1l* gene are correlated with intellectual disability, autism spectrum disorder, and obesity (2, 8). Studies suggest that *MYT1l* deficiency results in the aberrant activation of neuronal developmental programs, consequently impairing neuronal maturation and function[ (9). Moreover, the absence of *MYT1l* leads to an imbalance in neuronal proportions, predominantly affecting neuronal maturation processes. This developmental imbalance persists throughout the developmental timeline (10).

Mutations in the *MYT1l* gene are also implicated in schizophrenia and other neuropsychiatric disorders. Research has demonstrated that microdeletions in *MYT1l* are significantly associated with childhood-onset schizophrenia, indicating that *MYT1l* may contribute to the pathogenesis of these conditions (11). Additionally, *MYT1l* expression is linked to memory-related processes in the brain, influencing these processes by regulating the proliferation/differentiation switch of ID-bHLH factors (12).

In this case, both parents were healthy at the child's birth, with no history of intrauterine asphyxia or amniotic fluid contamination, and no family history of genetic disorders. Postnatally, the patient exhibited global developmental delay accompanied by manifestations of autism spectrum disorder. Based on the clinical presentation, whole-exome sequencing (WES) identified a *de novo* heterozygous missense variant in the *MYT1l* gene [c.1695G > T; p. (Arg565Ser)], which was confirmed by Sanger sequencing to segregate within the family according to genetic co-segregation principles. According to the ACMG/AMP guidelines, this variant was classified as “Likely Pathogenic” based on criteria PM6 (confirmed *de novo* status) and PM2 (absence in population databases). Despite the variant's classification as “Likely Pathogenic” and the need for functional validation, the significant overlap between the patient's phenotype and the established *MYT1l*-associated neurodevelopmental profile, in the absence of other plausible genetic findings, strongly implicates this variant as the most likely underlying cause. Therefore, integrating the compelling clinical and genetic evidence, a definitive diagnosis of Autosomal Dominant Intellectual disability 39 and Autism Spectrum Disorder was established.

The *MYT1l* variant at position 565 (p.Arg565Ser) (ID: 801647) lacks a definitive pathogenicity assignment in the ClinVar database, necessitating its evaluation using the PM6 and PM2 criteria. While our classification of “Likely Pathogenic” is robustly supported by *de novo* status and population frequency data, the application of other ACMG criteria warrants further discussion. the PS1 criterion (identical amino acid change as a known Pathogenic variant) could not be applied because this variant lacks independent basis for a “Pathogenic” or “Likely Pathogenic” designation. Similarly, the PP5 criterion (reported as Pathogenic by an authoritative source but with unpublished data) was also not met. Compared to the well-established loss-of-function mechanisms in *MYT1l*, the pathogenicity of this missense variant remains to be fully elucidated through functional studies. our case, combined with existing ClinVar submissions, adds to the body of evidence linking this specific variant to neurodevelopmental disorders and highlights the need for further evidence to strengthen its classification criteria.

After 20 months of structured rehabilitation training, the patient exhibited notable advancements in motor skills, including the ability to walk independently and run short distances, as well as enhancements in language abilities, demonstrated by the production of short phrases consisting of fewer than 10 words. Nevertheless, the patient continued to experience persistent deficits, such as diminished eye contact, inadequate response to auditory cues such as name calling, and repetitive head-banging behavior. Remarkably, in contrast to previously documented cases, this patient presented with normal findings on brain MRI, EEG results, and no history of seizure episodes.

We report a case of *MYT1l*-related MRD39, distinguished by pronounced global developmental delay and autism spectrum disorder, and characterized by a novel c.1695G > T missense variant that has not been previously documented. This finding broadens the current understanding of the mutation spectrum and phenotypic variability associated with *MYT1l,* thereby advancing insights into the genotype-phenotype correlations in conditions related to *MYT1l*.

## Patient perspective

The patient's legal guardian provided written informed consent for the publication of this case report.

## Data Availability

The original contributions presented in the study are included in the article/Supplementary Material, further inquiries can be directed to the corresponding author.
